# Metal Active‐Site Exposure via Ligand Engineering Boosts CO_2_‐to‐Ethylene Conversion on Cu_18_ Nanoclusters

**DOI:** 10.1002/advs.76879

**Published:** 2026-07-30

**Authors:** Ziqi Chen, Yang Zuo, Yu Zhu, Shuo Zhang, Along Ma, Shiyin Yang, Zhengmao Yin, Xiaoshuang Ma, Shuxin Wang

**Affiliations:** ^1^ College of Materials Science and Engineering Qingdao University of Science and Technology Qingdao People's Republic of China; ^2^ State Key Laboratory of Advanced Optical Polymer and Manufacturing Technology College of Chemistry and Molecular Engineering Qingdao University of Science and Technology Qingdao People's Republic of China

**Keywords:** active site, copper nanocluster, eCO_2_RR, intramolecular interactions, ligand engineering

## Abstract

In nano‐catalysts, constructing isostructural counterparts with precisely tunable surface and interface properties to unveil their structure‐activity relationships remains a significant challenge. Herein, we report the synthesis of two atomically precise, isostructural Cu hydride nanoclusters [Cu_18_H_17_(EtPP)_10_]^+^ (**Cu_18_‐1**) and [Cu_18_H_17_(TPP)_10_]^+^ (**Cu_18_‑2**), via ligand engineering. Although they share a similar metal‐core structure, their surface/ interface interactions exhibit marked differences. In the CO_2_ electroreduction reaction, **Cu_18_‐1** exhibits 70.59% selectivity for C_2_H_4_ with an industrial‐grade current density of −2.99 A·m^−2^, both of which are approximately twice those of **Cu_18_‐2**. This enhancement originates from the weaker intramolecular interactions in **Cu_18_‑1**, which facilitate phosphine‑ligand stripping under reaction conditions, thereby exposing more metal active sites and promoting C─C coupling. The mechanism is corroborated by post‑reaction mass spectrometry, theoretical calculations, and electrochemically active surface area measurements. This work provides fresh insights into the rational design of metal‐organic catalysts through ligand‑engineered regulation of intramolecular interactions.

## Graphical abstract

Through quantitative ligand stripping of [Cu_18_H_17_(PR_3_)_10_]^+^ nanoclusters via intramolecular interaction modulation, selectivity for CO_2_ electroreduction to C_2_H_4_ is significantly enhanced. The weakly coordinating ligand triggers dual‐ligand stripping (*FE*_C2H4_ = 70.59%), whereas the strongly coordinating ligand results in single‐ligand retention (*FE*_C2H4_ = 44.28%). This reveals the pivotal role of interfacial non‐covalent interactions in modulating the active sites of Cu catalysts.

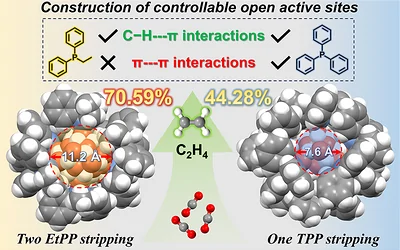

## 1 Introduction

Atomically precise ligand‐protected metal nanoclusters (NCs) have emerged as promising materials for electrocatalytic applications, owing to their well‐defined structures [1, 2, 3, 4, 5, 6], tunable physicochemical properties [7, 8, 9], and potential for elucidating catalytic mechanisms at the atomic level [10, 11, 12, 13, 14, 15, 16, 17, 18, 19, 20]. The coordination of ligands with metal cores critically governs surface interactions [21, 22, 23, 24, 25], electronic structures [26, 27, 28], and ultimately, catalytic performance [29, 30, 31, 32]. These atomically precise systems offer unique opportunities to identify active sites, establish structure‐activity relationships, and probe fundamental catalytic processes that remain elusive in larger nanoparticles [33, 34, 35, 36, 37, 38, 39, 40].

Electrochemical CO_2_ reduction (eCO_2_RR) represents a key technology for converting CO_2_ into value‐added multi‐carbon (C_2+_) products such as C_2_H_4_ and EtOH using renewable energy [41, 42, 43]. However, the identification of the active sites of cluster catalysts is considered crucial for investigating catalytic mechanisms. On one hand, ligand engineering provides a robust strategy for fabricating clusters that possess intrinsic open metal active sites, with interfacial cavities serving as catalytic centers, thus markedly enhancing both activity and selectivity. For instance, Cu_17_ NCs functionalized with dipropynyl‐modified N‐heterocyclic carbene ligands achieved 36% C_2_H_4_ selectivity [44]. Alternatively, an effective strategy involves the use of chelating ligands to design open cavity architectures. As we have previously reported, the exposure of a Cu_3_H_3_ triangular unit in AuCu_24_ NCs, achieved via a single di‐phosphine ligand, effectively promoted the generation of C_2_H_4_ (*FE* = 41.90%) [45]. On the other hand, the relatively weak interaction between the leaving ligand and the metal enables ligand stripping via electrical activation, yielding exposed metal active sites without compromising the structural integrity of the NCs. Taking Au_25_ NCs as an example, the removal of thiol ligands forms dethiolated Au sites, which markedly enhance the conversion efficiency of CO_2_ to CO [46]. Additional support comes from density functional theory (DFT) calculations, which reveal that the catalytically active sites in Cu_13_ and Cu_14_ NCs reside at the corner Cu atoms at the waist of the *C*_3_ symmetry axis, where dissociation of a single phosphine ligand has taken place [47]. Nevertheless, the packing of metal atoms in NCs formation is predominantly dictated by ligands, representing a fundamental constraint on the construction of open metal active sites via the aforementioned approaches. The key challenge remains the development of a universal and precise strategy to controllably fabricate such open active sites, while concurrently monitoring and elucidating ligand dissociation behavior.

To achieve ligand‐editable exfoliation, the precise modulation of intramolecular non‐covalent interactions, especially π···π stacking and C─H···π interactions, enables the construction of structurally stable catalytic active sites [48, 49, 50]. However, while strong π···π stacking interactions confer structural rigidity, they may restrict selective ligand dissociation; in contrast, moderate C─H···π interactions provide the necessary flexibility for dynamic ligand behavior. Therefore, ligand engineering on isostructural clusters offers a precise strategy for univariate regulation of interaction strength.

Herein, we investigate how ligand engineering modulates interfacial interactions and eCO_2_RR performance through controlled ligand stripping. We synthesized a pair of atomically precise, isostructural [Cu_18_H_17_(EtPP)_10_]^+^ and [Cu_18_H_17_(TPP)_10_]^+^ NCs (denoted as **Cu_18_‐1** and **Cu_18_‐2**, respectively), via ligand engineering by replacing triphenylphosphine (TPP, capable of engaging in multiple π···π interactions) with ethyldiphenylphosphine (EtPP). The two NCs share an essentially identical metal core framework, with ligand variation serving as the designed structural variable that modulates their intra‐cluster interaction networks. eCO_2_RR performance revealed that **Cu_18_‐1** exhibits superior selectivity toward C_2_H_4_, achieving a faradaic efficiency (*FE*) of 70.59% at −1.4 V_RHE_, which is approximately twice that of **Cu_18_‐2** (*FE*_C2H4_ = 44.28%). Correspondingly, at −1.4 V_RHE_, the current density for **Cu_18_‐1** (−298.62 mA·cm^−2^) substantially exceeds that of **Cu_18_‐2** (−120.24 mA·cm^−2^). Moreover, post‑reaction mass spectrometry and density functional theory (DFT) calculations collectively revealed that **Cu_18_‐1** is more prone to undergo double‐ligand elimination after eCO_2_RR, with its elimination energy barrier (4.33 eV) being significantly lower than that of the single‐ligand elimination of **Cu_18_‐2** (4.73 eV). Through in situ spectroscopy to monitor reaction intermediates and DFT calculations to investigate the potential energy pathways for C_2_H_4_ formation, the effective removal of multiple ligands enhances the exposure of Cu active sites, thereby promoting C─C coupling.

## 2 Results and Discussion

### 2.1 Synthesis and Characterization of Cu_18_H_17_ NCs

The inherent interactions between benzene rings on the ligand (e.g., π···π or C─H···π interactions) exerted a dual influence: they played a critical role in promoting crystal growth and stability via inter‐cluster interactions, yet overly strong intramolecular interactions could impede ligand dissociation during catalytic processes, limiting the exposure of active sites on the NCs. To address this issue, we replaced one phenyl ring in triphenylphosphine with an ethyl group, synthesizing a pair of isomeric Cu_18_ NCs (Figure S1). Briefly, a mixture of Cu^I^(MeCN)_4_BF_4_ and TPP was directly reduced using a MeOH solution of NaBH_4_ in a DCM/ MeOH mixture solvent. Upon completion, **Cu_18_‐2** was obtained as yellow block‐shaped crystals by slowly diffusing n‐hexane into the filtered DCM solution. Unfortunately, it failed to obtain EtPP‐protected Cu_18_ counterparts by the substitution of TPP with EtPP. However, the reaction conditions and the oxidation state of the metal salts play a crucial role in the formation of the Cu NCs. Notably, we successfully prepared the **Cu_18_‐1** NCs by directly reducing a mixture of Cu^II^(C_5_H_7_O_2_)_2_ and EtPP using an aqueous NaBH_4_ solution in a DCM/ MeOH mixture solvent, and obtained yellow block crystals after diffusion in n‐hexane/ DCM. This indicates that both the solvent reaction system and the valence state of the Cu salt play a crucial role in the formation of Cu NCs. The syntheses of both Cu_18_ NCs were readily scaled up 15‐fold without substantial loss in yield, affording **Cu_18_‐1** and **Cu_18_‐2** in 48.1% and 36.2% yields, respectively, and enabling gram‐scale preparation for subsequent studies (Figure S2). As illustrated in Figure 1a, this modification of **Cu_18_‐1** significantly reduced the plethora of intramolecular interactions present in the parent **Cu_18_‐2**, thereby creating more possibilities for exposing active sites in subsequent catalysis.

**FIGURE 1:**
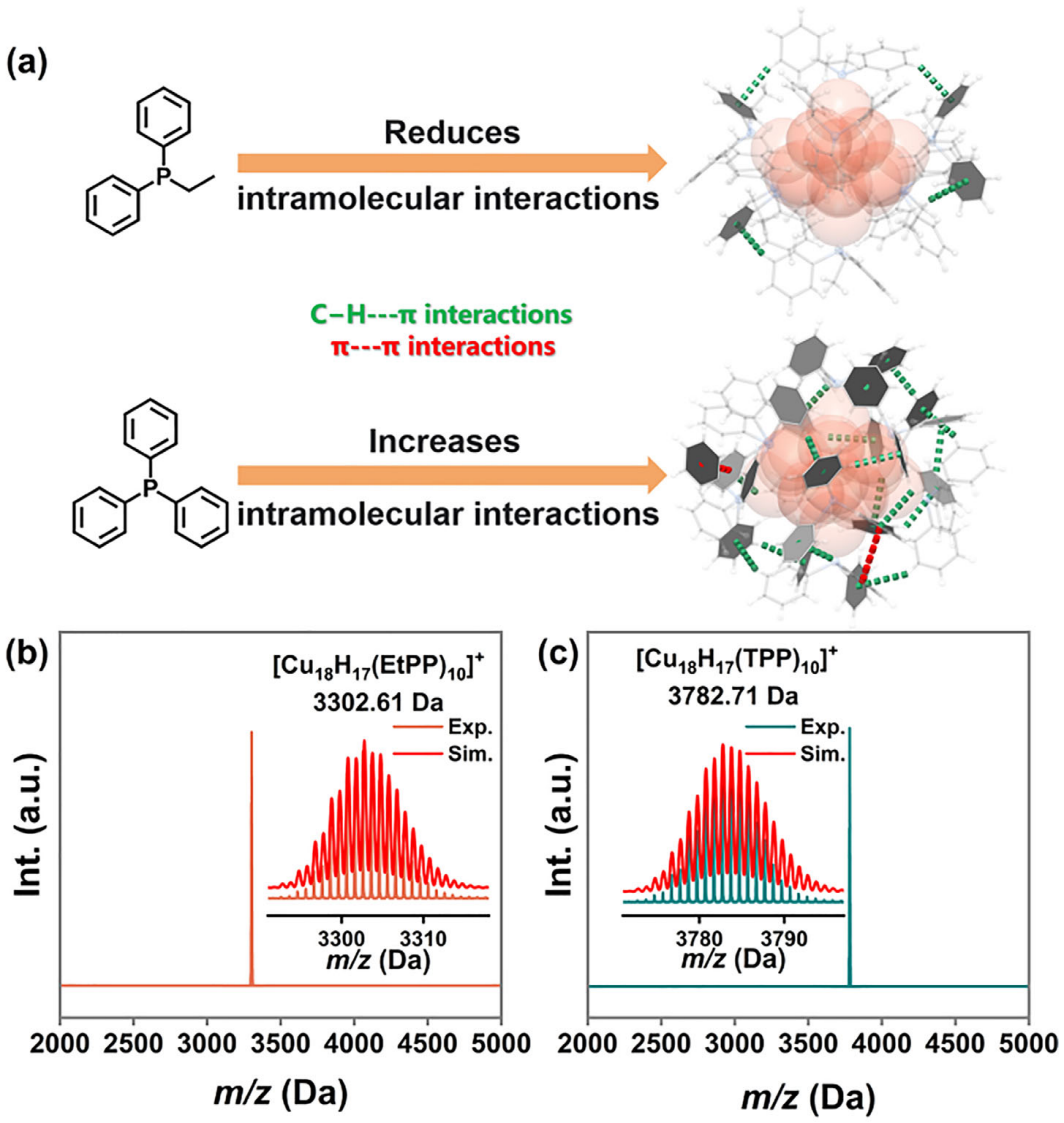
(a) Schematic of the modulation of intramolecular interactions in the Cu_18_ NCs via phosphine ligand π···π/ C─H···π interactions. ESI‐MS (in positive mode) of (b) [Cu_18_H_17_(EtPP)_10_]^+^ and (c) [Cu_18_H_17_(TPP)_10_]^+^ with experimental and simulated isotopic patterns.

The two Cu_18_ NCs were characterized by electrospray ionization mass spectrometry (ESI‐MS). As shown in Figure 1b,c, two distinct peaks were observed at *m/z* values of 3302.61 and 3782.71 Da, corresponding to the molecular formula of [Cu_18_H_17_(EtPP)_10_]^+^ (Calc. = 3302.78 Da, Δ = −0.17 Da) and [Cu_18_H_17_(TPP)_10_]^+^ (Calc. = 3782.78 Da, Δ = −0.07 Da), respectively. To unequivocally confirm the presence and quantity of hydride within the NCs core, **D‐Cu_18_‐1** was synthesized using NaBD_4_ instead of NaBH_4_. The ESI‐MS of **D‐Cu_18_‐1** exhibited a mass shift of the main peak to *m/z =* 3319.73 Da, with a Δ*m/z* upshift of 17.12 Da, which suggests the introduction of seventeen hydrides into the **Cu_18_‐1** framework (Figure S3). Despite sharing an identical metal core, the difference in surface ligands resulted in markedly distinct optical properties. The hydride environments in **D‐Cu_18_‐1** NCs were further probed by ^2^H‐NMR spectroscopy. The spectrum showed three resonances at 9.90, 2.43, and 0.66 ppm with an integrated ratio of 1.00:7.92:8.02, consistent with three hydride sets in an approximate 1:8:8 ratio and corroborating the assignment of 17 hydrides in **Cu_18_‐1** NCs (Figure S4). Crystallographic analysis, together with the retained characteristic aromatic ligand vibrations in the FT–IR spectra of crystalline and amorphous samples, supports distinct local ligand environments in the two Cu_18_ clusters and stronger intramolecular π···π/C─H···π interactions in TPP‐protected **Cu_18_‐2** than in EtPP‐protected **Cu_18_‐1** (Figure S5). As shown in Figure S6, the UV–vis absorption spectrum of **Cu_18_‐1** showed several broad absorption bands around 311, 370, and 485 nm, corresponding to an optical band gap (*E*_g_) of 2.33 eV. In contrast, **Cu_18_‐2** displayed a single broad absorption peak at 440 nm, with *E*_g_ of 2.47 eV. This discrepancy likely originated from the reduced intramolecular interactions in EtPP compared to TPP, which subsequently modulated the electronic structure of the NCs. The crystal morphology and elemental composition were investigated using scanning electron microscopy (SEM) and energy‐dispersive X‐ray spectroscopy (EDS, Figures S7 and S8). The metal valence states of the two Cu_18_ NCs were further analyzed by X‐ray photoelectron spectroscopy (XPS, Figure S9). As demonstrated in Figure S9b,e, the Cu 2p XPS revealed that the binding energy (BE) of the Cu 2p_3/2_ level for **Cu_18_‐1** (933.55 eV) is lower than that of **Cu_18_‐2** (934.05 eV), suggesting that replacing TPP in the coordination shell of the Cu_18_ NCs with EtPP leads to an increase in the electron density around the Cu atoms throughout the entire NCs. Since the Cu 2p_3/2_ XPS spectrum cannot unambiguously distinguish between Cu(0) and Cu(I), the Cu LMM Auger spectra were recorded, and the peaks observed at 570.53 eV for **Cu_18_‐1** and 570.63 eV for **Cu_18_‐2** confirm that all Cu atoms are predominantly in the Cu(I) oxidation state (Figure S9c,f).

### 2.2 Crystal Structure Analysis of Cu_18_H_17_ NCs

The molecular structures of the two Cu_18_ NCs were determined by single‐crystal X‐ray diffraction (SC‐XRD), revealing an identical metallic framework that differs only in the type of phosphine ligand (Figure 2a; Figures S10 and S11). Both Cu_18_ structures exhibited *C*_4_ symmetry and crystallize in the *P‐1* and *P2_1_/c* space groups, respectively, with their molecules packing in an “ABAB” sequence within the unit cell, stabilized by van der Waals forces and electrostatic interactions (Figures S12–S15). As shown in Figure 2b, the structural analysis of their metal kernels revealed a symmetrical six‐layer architecture (L1 to L6), where in the top (L1) and bottom (L6) layers each comprised a single Cu atom; the L2 and L5 layers consisted of four coplanar and bonded Cu atoms forming a square plane; and the L3 and L4 layers contained four coplanar yet non‐bonded Cu atoms. The Cu_18_ kernel was formed by the interpenetration of two Cu_9_ “pyramid” units: one from the L1, L2 and L3 layers, and the other generated by a 45° rotation of the first pyramid about the *C*_4_ axis from the L4, L5 and L6 layers.

**FIGURE 2:**
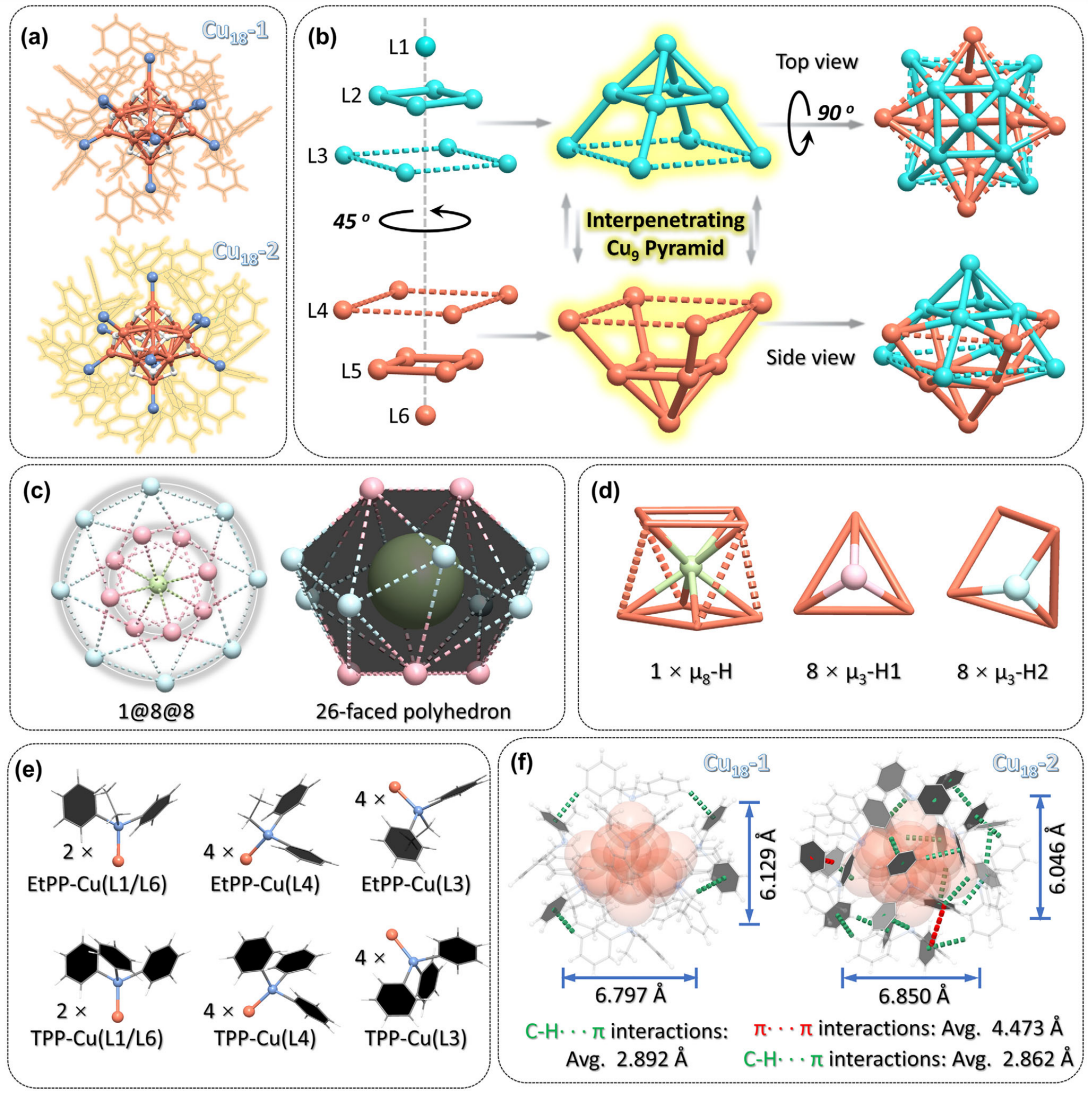
(a) Overall structure of **Cu_18_‐1** and **Cu_18_‐2**. (b) The distribution of Cu atoms in each layer and interpenetration of two Cu_9_ “pyramids” to form the Cu_18_ core. (c) The layered structure and twenty‐six polyhedral containing seventeen hydrogen atoms. (d) Cu─H coordination modes: 1 × *µ*_8_‐H, 8 × *µ*_3_‐H1, and 8 × *µ*_3_‐H2. (e) Spatial orientation comparison between the phosphine ligands of **Cu_18_‐1** and **Cu_18_‐2**. (f) Analysis of the monomeric dimensions and intramolecular interactions.

Seventeen hydrides within the Cu_18_ framework exhibit a 1@8@8 topological arrangement in a top‐down view, featuring one central hydride encapsulated by a 26‐faced polyhedron formed by the remaining sixteen hydrides (Figure 2c). Analysis of the hydride coordination modes revealed one octa‐coordinated (*µ*_8_‐H) and sixteen tri‐coordinated (*µ*_3_‐H) hydrides (Figure 2d). The *µ*_8_‐H hydride was anchored at the center of the cluster, coordinated to eight Cu atoms from the layers L2 and L5. The sixteen *µ*_3_‐H hydrides can be classified into two types: four *µ*_3_‐H1 hydrides bridge three Cu atoms within the L1/L2 layers to form a Cu_5_H_4_ moiety, a configuration replicated by another four *µ*_3_‐H1 hydrides in the L5/L6 layers (Figure S16), while the remaining eight *µ*_3_‐H2 hydrides were uniformly distributed between the L3 and L4 layers (Figure S17). On both Cu_18_ NCs, all ten phosphine ligands were situated within the L1/L6, L4, and L3 layers on the cluster surface, although the spatial orientations of their phenyl rings differ slightly (Figure 2e). Despite identical ligand coordination modes, the distinct phosphine ligands induce subtle structural distortions in the metal core. **Cu_18_‐1** exhibited a longitudinally elongated (6.129 Å) and laterally contracted (6.797 Å) core structure. In comparison, **Cu_18_‐2** had a longitudinal dimension of 6.046 Å (0.083 Å shorter than **Cu_18_‐1**) and a lateral dimension of 6.850 Å (0.053 Å longer than **Cu_18_‐1**, Figure 2f). Furthermore, comparative analysis revealed marginally shorter average bond lengths in **Cu_18_‐1** for both Cu─Cu (2.542 vs. 2.547 Å) and Cu─P (2.232 vs. 2.239 Å) relative to **Cu_18_‐2**, indicating that the fine structural difference between ligands reduced overall intramolecular interactions upon substituting TPP with EtPP (Figure S18). Specifically, **Cu_18_‐2** featured multiple π···π interactions (avg. distance: 4.473 Å) and shorter avg. C─H···π contact distances (2.862 Å) compared to **Cu_18_‐1** (2.892 Å). This structural modulation based on single‐benzene‐ring substitution on the phosphine ligand effectively reduced intramolecular interactions in Cu_18_ NCs, thereby conferring greater conformational flexibility and a more accessible reaction space for catalytic reactions.

### 2.3 Electrochemical CO_2_ Reduction

The eCO_2_RR performance of two Cu_18_ NCs supported on gas diffusion electrodes (GDE, 1 × 1 cm^2^) was evaluated via constant potential electrolysis (CPE) using a customized flow cell (Figure S19). The applied potential ranged from −0.9 to −1.6 V_RHE_ (the details of electrochemical measurements were provided in Supporting Information). Gas chromatography (GC) analysis identified four gaseous products (e.g., CO, CH_4_, C_2_H_4_, and H_2_) across all applied potentials without iR compensation, while the formation of liquid products (formate, EtOH, and CH_3_COOH) was confirmed by ^1^H‐NMR (Figures S20 and S21). Linear sweep voltammetry (LSV) analysis demonstrated that in a CO_2_‐saturated 0.50 M KOH and 0.50 M KCl mixed electrolyte, **Cu_18_‐1/GDE** exhibited significantly higher current densities than **Cu_18_‐2/GDE** over the entire potential range, indicating superior CO_2_ reduction activity (Figure 3a). In contrast, under an N_2_ atmosphere, the current densities for both catalysts were substantially lower than those in a CO_2_ atmosphere and showed negligible difference between them. The Tafel slopes for the two Cu_18_ catalysts were measured as 285 and 435 mV·dec^−1^, with **Cu_18_‐1/GDE** showing the lower value, suggesting more favorable reaction kinetics (Figure S22). Moreover, electrochemical impedance spectroscopy (EIS) measurements revealed that **Cu_18_‐1/GDE** exhibits a lower charge transfer resistance compared to **Cu_18_‐2/GDE**, in agreement with the LSV results (Figure S23). The two Cu_18_ catalysts exhibited distinct eCO_2_RR catalytic performance within the potential range of −0.9 to −1.6 V_RHE_ (Figure S24). Compared to **Cu_18_‐2/GDE**, **Cu_18_‐1/GDE** exhibited significantly higher selectivity toward C_2_H_4_ compared to the hydrogen evolution reaction (HER) and other carbon‐containing products within the potential range of −1.1 to −1.6 V_RHE_ (Figure S25). Specifically, the *FE* of C_2_H_4_ for **Cu_18_‐1/GDE** increased steadily from −0.9 V_RHE_, reaching a maximum of 70.59% at −1.4 V_RHE_, which is nearly twice that of **Cu_18_‐2/GDE** (*FE*_C2H4_ = 44.28%, Figure 3b). As presented in Figure 3c, the partial current density (*j*) for C_2_H_4_ on **Cu_18_‐1/GDE** reached a maximum of −298.62 mA·cm^−2^ at −1.4 V_RHE_, which was more than double that of **Cu_18_‐2/GDE** (*j* = −120.24 mA·cm^−2^). The high selectivity of **Cu_18_‐1/GDE** for C_2_H_4_ resulted in very low *FEs* and partial current densities (*j*) for other C‐containing products (CO, formate, CH_4_, EtOH, and CH_3_COOH) and H_2_ (Figures S26 and S27).

**FIGURE 3:**
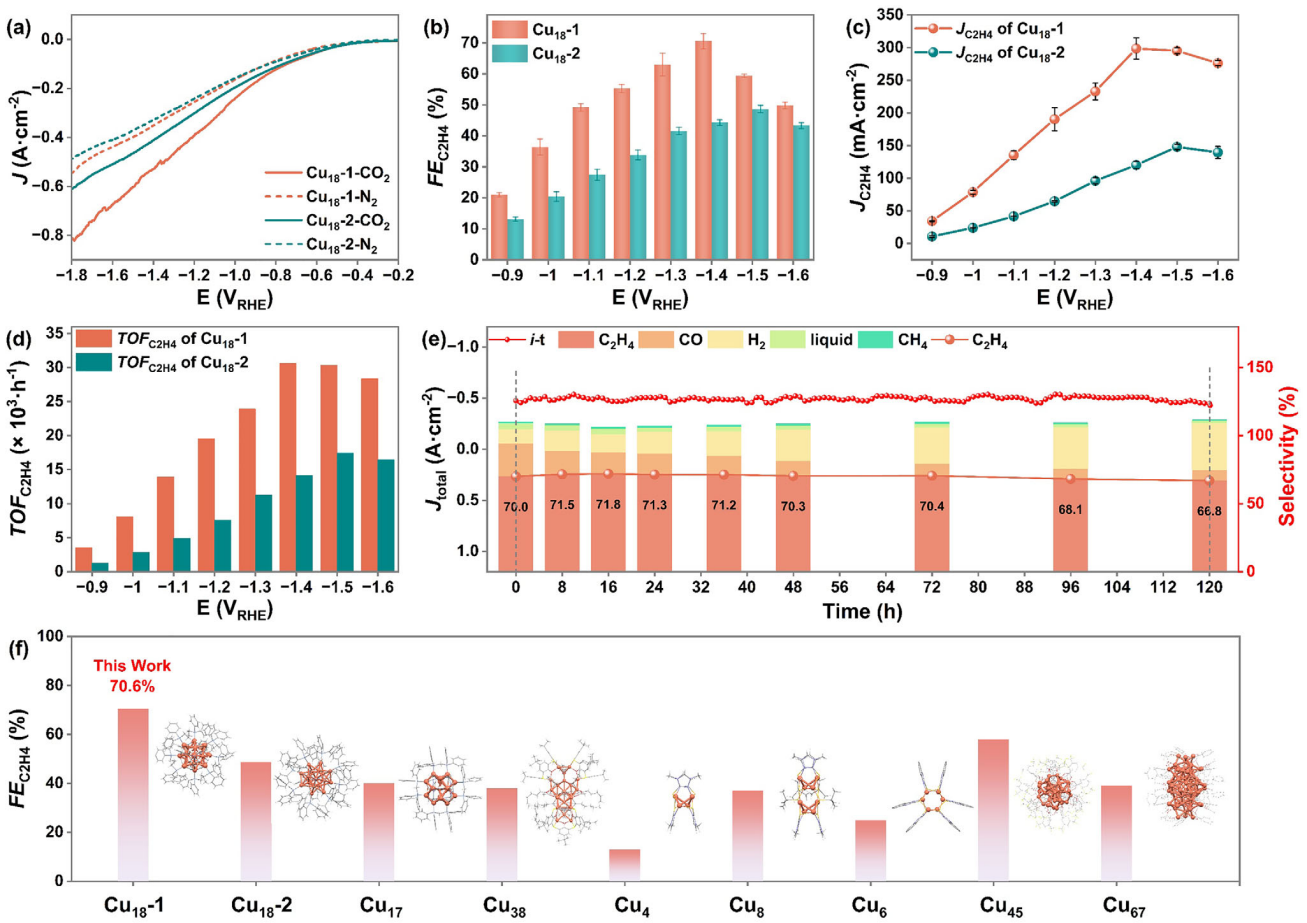
(a) LSV curves of **Cu_18_‐1** and **Cu_18_‐2** under CO_2_ and N_2_ saturation. Comparison of (b) *FE*, (c) *J*, and (d) *TOF* of C_2_H_4_ production between **Cu_18_‐1** and **Cu_18_‐2**. (e) Long‐term stability test of C_2_H_4_ production on **Cu_18_‐1** over 120 h. (f) Comparison of C_2_H_4_ selectivity among recently reported Cu NCs catalyst.

To rule out a dominant counterion effect, an anion‐exchange control was conducted by replacing the Cl^−^ counterion in **Cu_18_‐1** with BF_4_^−^, affording [Cu_18_H_17_(EtPP)_10_]BF_4_ (**Cu_18_‐1‐BF_4_**). SC‐XRD confirmed that **Cu_18_‐1‐BF_4_** retains the Cu_18_H_17_ core structure after counterion exchange, while its molecular composition was further verified by UV–vis absorption spectroscopy, ESI‐MS, and SEM‐EDS (Figures S28–S30). Under identical eCO_2_RR conditions, **Cu_18_‐1‐BF_4_** displayed catalytic activity and C_2_H_4_ selectivity comparable to those of **Cu_18_‐1**, but markedly different from those of **Cu_18_‐2** (Figure S31). These results indicate that the difference in eCO_2_RR performance is not primarily governed by the counterion, but instead arises from the distinct ligand environments and the associated ligand‐regulated intracluster interactions. Furthermore, the mass spectrum of ^13^C‐labeled CO_2_ clearly shows peaks for ^13^CH_4_ (*m/z* = 17 Da), ^13^CO (*m/z* = 29 Da), and ^13^C_2_H_4_ (*m/z* = 30 Da), fully demonstrating that the carbon‐containing products in eCO_2_RR originate from CO_2_ (Figure S32).

Given the notable disparity in their *FE*_C2H4_, the intrinsic activity of the two catalysts was further quantified by calculating the turnover frequency (*TOF*) for C_2_H_4_ production. As shown in Figure 3d, the *TOF* values for **Cu_18_‐1/GDE** were significantly higher than those for **Cu_18_‐2/GDE** across all tested potentials. At the optimal potential of −1.4 V_RHE_, **Cu_18_‐1/GDE** exhibited a *TOF* value of 30.62 × 10^3^ h^−1^, which was about twice that of **Cu_18_‐2/GDE** (14.14 × 10^3^  h^−1^). This result indicated that each active site on **Cu_18_‐1/GDE** can convert more CO_2_ molecules to C_2_H_4_ per unit time, underscoring its superior intrinsic catalytic activity. Furthermore, the double‐layer capacitance (*C*_dl_) of **Cu_18_‐1/GDE** (5.28 mF·cm^−2^) compared to **Cu_18_‐2/GDE** (4.75 mF·cm^−2^) reflected its larger electrochemically active surface area and superior charge transfer capability (Figure S33).

Catalytic stability of the Cu_18_ clusters was evaluated in a flow cell (Figure 3e; Figure S34). During 120 h electrolysis, **Cu_18_‐1/GDE** and **Cu_18_‐2/GDE** maintained stable current densities of −0.50 and −0.45 A·cm^−2^, respectively, with *FE*_C2H4_ values remaining at 66.8% and 45.0%. The structural stability of Cu_18_ clusters during eCO_2_RR was further evaluated by post‐reaction characterizations. ^1^H‐NMR characterization of the DCM extracts from the post‐reaction working electrode indicated slight cluster decomposition and aggregation, while ICP analysis quantified the decomposition loss of Cu_18_ clusters as only 2.19% (Figure S35). After 120 h electrolysis, ESI‐MS confirmed the retention of the Cu_18_ signal without further ligand stripping, and ICP analysis showed a decomposition loss of 3.73%, indicating that 96.27% of the Cu_18_ clusters remained preserved (Figure S36). In addition, the cluster‐free GDE after DCM extraction showed only HER activity, whereas redeposition of the recovered clusters restored the eCO_2_RR selectivity to near‐original levels (Figure S37). Comparative XPS and HR‐TEM of the preserved Cu_18_ before and after catalysis revealed negligible changes in the oxidation state and overall size of the NCs (Figures S38 and S39). These results demonstrate that Cu_18_ largely maintains its structural integrity during long‐term eCO_2_RR, and the catalytic activity mainly originates from Cu_18_ NCs rather than trace decomposition products. **Cu_18_‐1** was further benchmarked against reported Cu NC catalysts and representative Cu‐based catalysts, showing an *FE*_C2H4_ above 70% under comparable flow‐cell conditions (Figure 3f; Table S3).

### 2.4 Mechanistic Investigations of eCO_2_RR

To further elucidate the behavior of the phosphine (‐PR_3_) ligands on the surface of NCs during the catalytic process, we performed ESI‐MS measurements of **Cu_18_‐1** and **Cu_18_‐2** before and after the eCO_2_RR. As indicated in Figure 4a,b, both catalysts exhibited only a single molecular ion peak before eCO_2_RR. After electrolysis, two new feature peaks appeared at *m*/*z* 2874.46 and 3088.53 Da for **Cu_18_‐1**, corresponding to [Cu_18_H_17_(EtPP)_8_]^+^ (Calc. = 2874.59 Da, Δ = −0.13 Da) and [Cu_18_H_17_(EtPP)_9_]^+^ (Calc. = 3088.68 Da, Δ = −0.15 Da), are attributed to species have removed one and two ‐PR_3_ ligands, respectively. In contrast, for **Cu_18_‐2** after eCO_2_RR, only the species that had lost one ‐PR_3_ ligand at *m*/*z* 3520.66 Da, assigned to [Cu_18_H_17_(TPP)_9_]^+^ (Calc. = 3520.69 Da, Δ = 0.03 Da). Together with the crystallographically resolved intracluster interactions, these ESI‐MS results indicate that reductive ligand stripping exposes distinct Cu active sites, leading to divergent eCO_2_RR behavior (Figure S40). To further elucidate the thermodynamic origins of the distinct ligand‐stripping behaviors of the two catalysts, DFT calculations were performed for **Cu_18_‐1** and **Cu_18_‐2** under an identical computational setup to compare their relative energetics (Figure 4c). The results revealed that the Cu─P bond dissociation energies (*Δ*G_Cu‐P_) for removing a single ‐PR_3_ ligand in **Cu_18_‐1** (2.15 eV) were much lower than those in **Cu_18_‐2** (*Δ*G_Cu‐p_ = 4.73 eV). Notably, the energy required to remove two ‐PR_3_ ligands from **Cu_18_‐1** is 4.33 eV, which was even lower than that for removing just one ligand from **Cu_18_‐2**, and markedly lower than that for removing two ligands from **Cu_18_‐2** (6.49 eV). These findings could be rationalized by the weakened interfacial interactions in **Cu_18_‐1**, which facilitate ligand dissociation.

**FIGURE 4:**
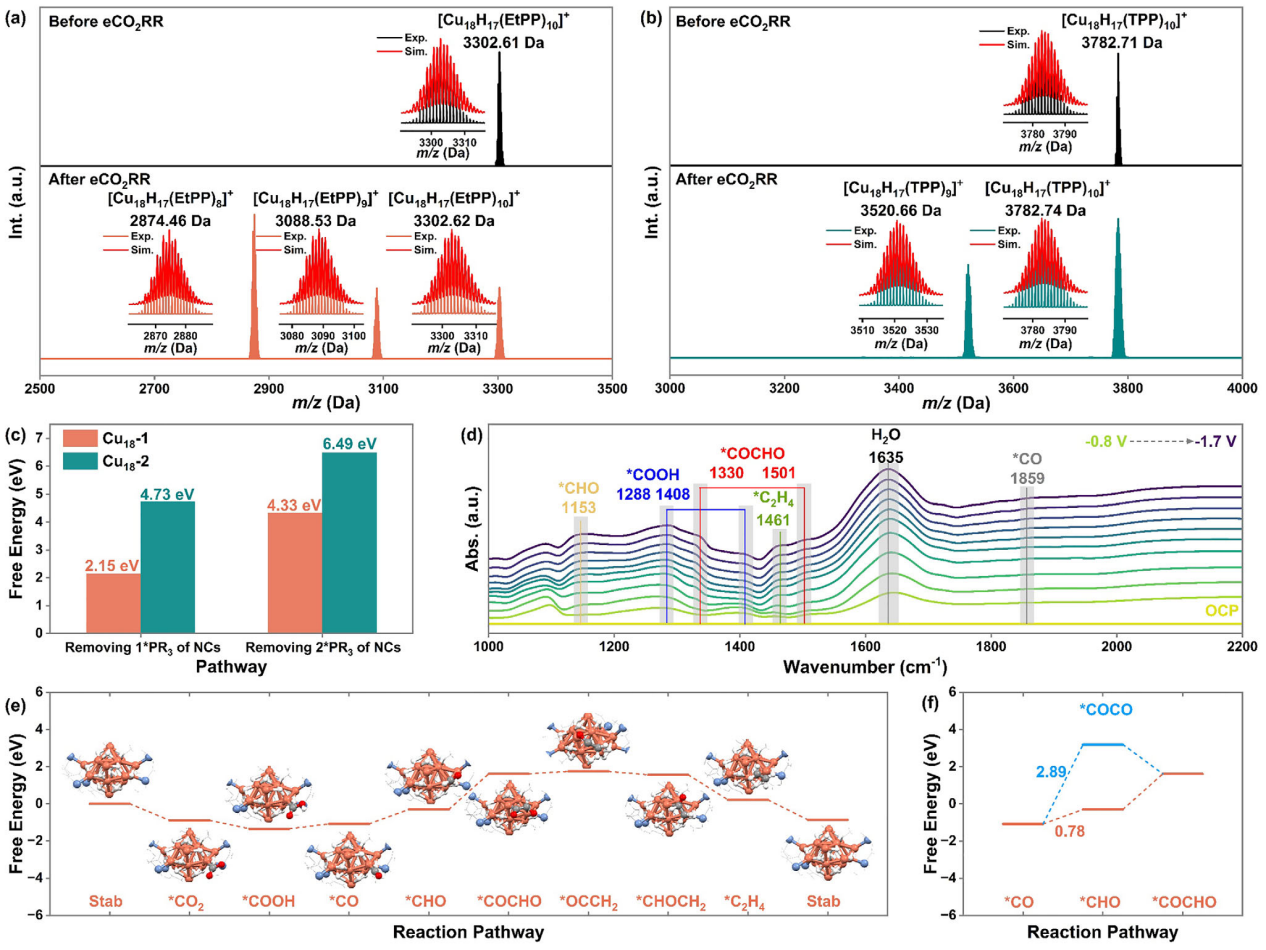
ESI‐MS of (a) **Cu_18_‐1** and (b) **Cu_18_‐2** before and after eCO_2_RR (Insets: the experimental and calculated isotopic patterns). (c) Cu‐P dissociation energy of removing one or two ‐PR_3_ ligands from **Cu_18_‐1** and **Cu_18_‐2**. (d) In situ ATR‐SEIRAS spectroscopy of the **Cu_18_‐1** collected from −0.8 to −1.7 V_RHE_ during eCO_2_RR. OCP represents the open‐circuit potential. (e) Gibbs free energy (*Δ*G) diagrams of the CO_2_ to C_2_H_4_ pathway on **Cu_18_‐1**. (f) the *Δ*G comparison of ^*^CO to ^*^CHO/^*^CO‐^*^CO. The asterisk (^*^) denotes adsorbed species on active sites.

To further elucidate the origin of the high eCO_2_RR selectivity toward C_2_H_4_ on **Cu_18_‐1**, in situ infrared spectroscopy and density functional theory (DFT) calculations were employed. We first performed in situ ATR‐SEIRAS to identify the eCO_2_RR intermediates. As shown in Figure 4d, a distinct broad peak was observed at 1635 cm^−1^, corresponding to the O─H bending vibration of adsorbed H_2_O. The peaks located at 1288 and 1408 cm^−1^ are assigned to the C─O stretching and symmetric vibrations of ^*^COOH [51], a key intermediate of ^*^CO, respectively. The peak at 1153 cm^−1^ was attributed to ^*^CHO [52]. Notably, these peaks at 1330 and 1501 cm^−1^ correspond to ^*^COCOH [53], which was a key intermediate in the C‐C coupling reaction that leads to the formation of C_2_H_4_. DFT calculations were performed to reveal the catalytic pathway of CO_2_ to C_2_H_4_ on the ligand‐dissociated **Cu_18_‐1**. The Gibbs free energy (*Δ*G) profile revealed the spontaneity of the process from the initial state to ^*^COOH, as evidenced by continuously decreasing energy barriers (Figure 4e). Subsequently, the energy barrier for the protonation of ^*^CO to ^*^CHO (0.78 eV) was significantly lower than that for the C─C coupling to form ^*^COCO (2.89 eV, Figure 4f). Therefore, ^*^CO preferentially undergoes protonation to form ^*^CHO, which then couples with another ^*^CO to yield the key C_2_H_4_ intermediate, ^*^COCHO, with a barrier of 1.91 eV. Further protonation steps ultimately led to the formation of C_2_H_4_. Consequently, the predominant pathway for CO_2_‐to‐C_2_H_4_ conversion on **Cu_18_‐1** could be delineated as: ^*^CO_2_(g) → ^*^COOH → ^*^CO → ^*^CHO → ^*^COCHO → ^*^OCCH_2_ → ^*^CHOCH_2_ → ^*^ +C_2_H_4_(g).

## 3 Conclusion

In summary, precise modulation of interfacial non‐covalent interactions in Cu_18_ NCs enabled targeted control of active site regions, achieving highly efficient electrochemical CO_2_‐to‐C_2_H_4_ conversion. The **Cu_18_‐2** NCs assembled with TPP ligands exhibit multiple intermolecular C─H···π and π···π interactions on their surface. In contrast, the **Cu_18_‐1** cluster formed with EtPP ligands shows a marked reduction in such surface interactions. This difference facilitates the stripping of two phosphine ligands from **Cu_18_‐1** during electrocatalysis, thereby exposing a larger active surface area. Consequently, at an applied potential of −1.4 V_RHE_, **Cu_18_‐1** delivers a *FE*_C2H4_ of 70.59% and a *j*_C2H4_ of −298.62 mA·cm^−2^, which are nearly double the values obtained for **Cu_18_‐2** (*FE*_C2H4_ = 44.28%, *j*_C2H4_ = −120.24 mA·cm^−2^). The pathway for ethylene formation is elucidated through in situ ATR‐SEIRAS and DFT calculations. Furthermore, post‐reaction ESI‐MS and theoretical calculations confirm that weaker intramolecular interactions promote more extensive ligand stripping. This work not only deepens the fundamental understanding of how intramolecular interactions can be manipulated to tune catalytic outcomes but also provides a feasible strategy, using ESI‐MS, to verify ligand stripping post‐reaction.

[CCDC for **Cu_18_‐1** and **Cu_18_‐1‐BF_4_** are 2451470 and 2550710, respectively, which contain the supplementary crystallographic data for this paper. These data can be obtained free of charge from The Cambridge Crystallographic Data Centre via www.ccdc.cam.ac.uk/data_request/cif].

## Author Contributions

**Ziqi Chen**: methodology, conceptualization, investigation, validation, formal analysis, data curation, Writing – original draft, visualization. **Shuxin Wang**: writing – review and editing, writing – original draft, conceptualization, methodology, resources. **Shiyin Yang**: visualization, data curation. **Xiaoshuang Ma**: conceptualization, writing – original draft, writing – review and editing, methodology. **Yu Zhu**: validation, visualization, formal analysis. **Shuo Zhang**: data curation, validation. **Along Ma**: validation, data curation. **Yang Zuo**: investigation, methodology, validation, formal analysis. **Zhengmao Yin**: writing – original draft, writing – review and editing.

## Conflicts of Interest

The authors declare no conflicts of interest.

## Supporting information




**Supporting File**: advs76879‐sup‐0001‐SuppMat.docx.

## Data Availability

The data that support the findings of this study are available from the corresponding author upon reasonable request.
