# Supplementary material for: Metal Active‐Site Exposure via Ligand Engineering Boosts CO2‐to‐Ethylene Conversion on Cu18 Nanoclusters
Source: Adv Sci (Weinh). 2026 Jul 30;13(59):e76879. doi: 10.1002/advs.76879 (PMC13423505; doi:10.1002/advs.76879)
Supplement: Supplementary file 1 [file ADVS-13-e76879-s001.docx]

Supporting Information

**Metal Active-Site Exposure via Ligand Engineering Boosts CO_2_-to-Ethylene Conversion on Cu_18_ Nanoclusters**

Ziqi Chen,^a^ Yang Zuo,^a^ Yu Zhu,^a^ Shuo Zhang,^a^ Along Ma,^b^ Shiyin Yang,^a^ Zhengmao Yin,*^,a^ Xiaoshuang Ma,*^,a^ and Shuxin Wang*^,a,b^

^a^ College of Materials Science and Engineering, Qingdao University of Science and Technology, Qingdao 266042, P. R. China.

E-mail: yzm198752@163.com, xiaoshuang_ma@qust.edu.cn

^b^ State Key Laboratory of Advanced Optical Polymer and Manufacturing Technology, College of Chemistry and Molecular Engineering, Qingdao University of Science and Technology, Qingdao 266042, P. R. China.

E-mail: shuxin_wang@qust.edu.cn

**Table of Contents**

[Section 1. Experimental Procedures	3](#_Toc233356850)

[Synthesis.	3](#_Toc233356851)

[Characterization.	3](#_Toc233356852)

[Electrospray ionization mass spectrometry.	4](#_Toc233356853)

[X-ray crystallography.	4](#_Toc233356854)

[In situ ATR-FTIRS measurements.	4](#_Toc233356855)

[Electrochemical measurements.	5](#_Toc233356856)

[Comprehensive characterization of Cu_18_ NCs after eCO_2_RR	6](#_Toc233356857)

[DFT calculations.	6](#_Toc233356858)

[Section 2. Supporting Figures	8](#_Toc233356859)

[Section 3. Supporting Table	32](#_Toc233356860)

Section 1. Experimental Procedures

Synthesis.

**Chemicals and Materials.** All reagents were commercially available and used without further purification. tetrakis(acetonitrile)copper(I) tetrafluoroborate (Cu^I^(MeCN)_4_BF_4_, 98% metals basis), Cupric(II) acetylacetonate (Cu^II^(C_5_H_7_O_2_)_2_, 99% metals basis), ethyldiphenylphosphine (EtPP, 99%), triphenylphosphine (TPP, 99%), sodium borohydride (NaBH_4_, 98%), methanol (MeOH, HPLC grade), dichloromethane (DCM, HPLC), and *n*-hexane (Hex, HPLC), were used to carry out the experiments. All glassware was thoroughly cleaned with aqua regia (V_HCl_: V_HNO3_ = 3: 1), rinsed with copious pure water, and then dried in an oven prior to use.

**Synthesis of Cu_18_H_17_(EtPP)_10_Cl NCs (Cu_18_-1)**. At room temperature, Cu^II^(C_5_H_7_O_2_)_2_ (72 mg, 0.275 mmol) was dissolved in a mixed solvent of 15 mL DCM and 5 mL MeOH (3: 1, v/ v), which was stirred for 30 minutes. Subsequently, under vigorous stirring, EtPP (77 μL, 0.38 mmol) was added. Then, 2 mL of a freshly prepared NaBH_4_ (60 mg) in H_2_O was directly poured into the reaction mixture. The reaction proceeded for a duration of 5 hours, after which the organic phase was washed multiple times with Hex. Finally, the product was dissolved in DCM and diffused into Hex. After approximately 3 days at −4 ^o^C, yellow block-shaped crystals were obtained (yield: ~46% based on Cu salt).

**Synthesis of Cu_18_H_17_(TPP)_10_BF_4_ NCs (Cu_18_-2)**. At room temperature, Cu^I^(MeCN)_4_BF_4_ (63 mg, 0.2 mmol) was dissolved in a mixed solvent of 10 mL DCM and 10 mL MeOH (1: 1, v/ v), followed by the addition of TPP (66 mg, 0.25 mmol), which was stirred for 20 minutes. Then, under vigorous stirring, 2 mL of a freshly prepared NaBH_4_ (60 mg) in MeOH was directly poured into the reaction mixture. The reaction proceeded for a duration of 4 hours; after which the organic phase was washed multiple times with Hex. Finally, the product was dissolved in DCM and diffused into Hex. After approximately 3 days at −4 ^o^C, yellow block-shaped crystals were obtained (yield: ~35% based on Cu salt).

**Synthesis of Cu_18_D_17_(EtPP)_10_Cl and Cu_18_D_17_(TPP)_10_Cl NCs.** The deuterated **Cu_18_** nanoclusters were prepared using the same procedure as the **Cu_18_-1 and Cu_18_-2** nanoclusters via replacing NaBH_4_ with NaBD_4_.

Characterization.

The UV-vis absorbance spectra (UV-vis) measurements were recorded on an Agilent Cary 5000 UV-vis-NIR spectrophotometer. All crystal samples were dissolved in DCM for spectroscopic analysis. The X-ray photoelectron spectroscopy (XPS) measurements were performed on ESCALAB XI+ configured with a monochromated Al K_α_ (1486.8 eV) 150W X-ray source, 0.5 mm circular spot size, a flood gun to counter charging effects, and the analysis chamber base pressure lower than 1×10^−9^ mbar, data were collected with FAT = 20 eV. The crystals of NCs were adhered to packaging tape for testing, and a small amount of carbon was added to assist with data correction. The scanning electron microscopy with energy-dispersive X-ray spectroscopy (SEM-EDS) measurements were conducted on HITACHI Regulus 8100. The crystals of NCs were tested by sticking on a conductive adhesive with an accelerating voltage of 0.1 to 30 kV. Proton nuclear magnetic resonance (^1^H-NMR and ^2^H-NMR) spectra measurements were performed using a Bruker Avance spectrometer operating at 500 MHz.

Electrospray ionization mass spectrometry.

ESI-MS measurements were carried out on a Bruker micro TOF-Q system in positive or negative ion mode in the range *m*/z = 1000-6000. The samples were dissolved in DCM/ MeOH (V/ V = 1/ 3, Ca. 1 mg·mL^−1^). The data analyses of mass spectra were performed based on the isotope distribution patterns using Compass Data Analysis software (Version 4.4).

X-ray crystallography.

Single crystal X-ray diffraction (SC-XRD) measurements were carried out on a Bruker D8 Quest at 150 K, using a Mo K_α_ radiation (λ = 0.71073 Å). The structure was solved by intrinsic phasing and refined with full-matrix least squares on *F*^2^ using the SHELXTL software package. All non-hydrogen atoms were refined anisotropically, and all the hydrogen atoms were set in geometrically calculated positions and refined isotopically using a riding model. Detailed crystal data for **Cu_18_-1** and **Cu_18_-1-BF_4_** NCs are given in Table S1-2, CCDC for **Cu_18_-1** and **Cu_18_-1-BF_4_** are 2451470 and 2550710, respectively, which include the supplementary crystallographic data for this paper. These data are provided free of charge by the Cambridge Crystallographic Data Centre.

In situ ATR-FTIRS measurements.

*In situ* attenuated total reflection surface-enhanced infrared radiation absorption spectroscopy (ATR-SEIRAS) was conducted using a Nicolet iS50 FTIR spectrometer equipped with a narrow band MCT-A detector and an *in-situ* IR optical accessory at an incidence angle of ca. 30°. IR spectra were collected with unpolarized IR radiation at a spectral resolution of 8 cm^−1^. All spectra are shown in absorbance, defined as −log(R/R_0_), where R and R_0_ represent the sample and reference single-beam spectra, respectively. The DONGHUA DH7001B electrochemistry workstation (Jiangsu Donghua Analytical Instrument Co., Ltd) was used for potential control and current measurement. The electrochemical setup comprised a germanium single crystal as the working electrode, an Ag/AgCl reference electrode, and a platinum sheet as the counter electrode. A catalyst loading of 5 mg was dispersed in 1 mL DCM with 20 μL of 0.05 % Nafion solution and coated onto the germanium crystal surface. Before measurement, CO_2_ was purged into the electrolyte at a flow rate of 20 sccm for 30 minutes. Real-time spectra were recorded under chronoamperometric conditions, with triplicate tests conducted at each applied potential (ranging from –0.8 V to –1.7 V_RHE_).

Electrochemical measurements.

The catalyst sample was prepared by loading Cu_18_ NCs onto Ketjen Carbon (C) at a mass ratio of 1:1 (5 mg NC and 5 mg C), which was subsequently dispersed in isopropyl alcohol at a concentration of 2.5 mg·mL⁻¹ under sonication for 10 mins. The final catalyst ink was prepared by thoroughly mixing 1 mL of catalyst suspension with 20 μL of Nafion (5 wt.%). Working electrodes were fabricated by casting 40 μL of this solution (with a NCs loading of 0.5 mg·cm^−2^) onto the effective contact area (2 × 0.5 cm^2^) of gas diffusion electrode (GDE), which was subsequently dried at room temperature.

Electrochemical CO_2_ reduction was conducted at ambient environment on the DONGHUA DH7001B electrochemical workstation (Jiangsu Donghua Analytical Instrument Co., Ltd). Linear sweep voltammetry (LSV) was performed from −0.2 to −2.2 V_RHE_ at a scan rate of 10 mV·s^−1^. The electrochemical performance of three catalysts was tested in a custom-designed flow cell reactor. Hg/HgO in 1.0 M KOH saturated aqueous electrolyte was employed as the reference electrode. During the measurement process, the flow rate of CO_2_ into the cathode GDE was 20 mL·min^−1^, and the flow rate of the cathode liquid was 40 rpm, which was controlled by a peristaltic pump (Cole-Parmer). 0.5 M KOH and 0.5 M KCl mixed solution (pH = 13.7) used as the electrolyte. Electrode potentials measured on the Hg/HgO scale (E_Hg/HgO_) were converted into the reversible hydrogen electrode (RHE) scale using the following equation:

$$\text{E}_{\text{(RHE)}}\text{ =}\text{ E}_{\text{(Hg/HgO)}}\text{ + 0.098 + 0.059 × pH}\text{ (S1)}$$

To identify and quantify the gaseous products, the output of the gas flow from the cathode chamber was directed into a gas chromatograph instrument (GC, RUINENG 3900 Plus), which was purged for 30 mins prior to the test. The liquid products were analyzed using a Bruker AVANCE III 400 MHz NMR instrument with DMSO as an internal standard. The faradaic efficiency (*FE*_X_) and partial current density (*j*_X_) of the reduction products such as H_2_, CO, CH_4_, and C_2_H_4_ (denoted X below) were calculated as follows:

$$\text{FE}_{\text{X}}\text{ = }\frac{\left( \text{N}_{\text{i}}\text{ × n × F} \right)}{\text{Q}_{\text{t}}} \text{(S2)}$$

$$\text{j}_{\text{X}}\text{ = }\frac{\text{FE}_{\text{X}}\text{ × }\text{Q}_{\text{t}}}{\text{t × }\text{Area}} \text{(S3)}$$

where

*Q_t_* = total charge consumed in the electrochemical reaction

*N_i_* = the number of moles of the product (measured GC)

*n* = the number of electrons transferred in the elementary reaction (2 for CO, H_2,_ and formate; 8 for CH_4_ and CH_3_COOH; 12 for C_2_H_4_ and EtOH)

*F* = the Faradaic constant (96485 C·mol^−1^)

*t* = reaction time (s)

Area = geometry area of the electrode (1 cm^2^)

Comprehensive characterization of Cu_18_ NCs after eCO_2_RR

After eCO_2_RR, the working electrode was removed, gently rinsed with deionized water, and dried at room temperature. The post-reaction electrode was then thoroughly immersed in excess DCM to extract soluble Cu_18_ species from Cu particles formed by cluster decomposition/aggregation. The obtained DCM extract was analyzed by ^1^H-NMR and compared with that of an equivalent amount of pristine Cu_18_. The DCM-extracted electrode was further acid-digested, diluted to a defined volume, and analyzed by ICP. For control experiments, the DCM extract was drop-cast onto a fresh GDE to prepare an extract-reconstructed electrode, while the residual Cu/GDE after extraction was directly used as a working electrode. Both electrodes were tested under identical eCO_2_RR conditions, and the product distribution, Faradaic efficiency, and current density were recorded.

DFT calculations.

Periodic DFT calculations were performed with the VASP code using the Perdew-Burke-Ernzerhof exchange-correlation function of the generalized gradient approximation (GGA). Projector augmented wave (PAW) pseudopotential was used to describe core-valence interactions. The plane-wave cutoff energy was set to 400 eV. The Brillouin zone of the reciprocal space was sampled with the Γ-centred Monkhorst-Pack scheme, the k-point mesh was set to 1 × 1 × 1 for the geometry optimization. The energy convergence of the self-consistent iteration reached 10^−5^ eV per atom and the Hermann-Feynman force convergence 0.02 eV Å^–1^, ensuring that the structure optimization process converged to a stable structure. To maintain a consistent basis for comparison, an identical simplified vacuum model was used for both cluster models without explicit water molecules or electrolyte ions; hence, the calculations are intended to resolve relative trends in ligand stripping and reaction energetics rather than to provide absolute values for a fully solvated alkaline electrochemical interface.

The computational hydrogen electrode (CHE) model was used to calculate the Gibbs free energy of the electrochemical and non-electrochemical elementary steps involved in ECR, defined as:

$$\text{Δ}\text{G}\text{ = }\text{Δ}\text{E}\text{ }-\text{ }\text{T}\text{Δ}\text{S}\text{ + }\text{Δ}\text{ZPE}$$

Here, ΔE is the difference in the total energy of the reaction of the elementary steps as calculated by the DFT in vacuum. TΔS and ΔZPE are the entropy and zero-point energy changes at 298.15 K, respectively.

Section 2. Supporting Figures

**Figure S1.** Photographs of the synthesis process of (a) **Cu_18_-1** and (b) **Cu_18_-2**.

**Figure S2.** Weighed masses of isolated pure (a) **Cu_18_-1** and (b) **Cu_18_-2** NCs obtained from a single large-scale synthesis. In this case, 1 g of Cu salt was used.

**Figure S3.** ESI-MS profiles in positive mode of **H-Cu_18_-1** and **D-Cu_18_-1**.

**Figure S4. D-Cu_18_-1** in CHCl_3_ was analyzed by ^2^H-NMR. Note: The peak at 5.32 ppm corresponds to CD_2_Cl_2_ used for chemical shift calibration; the peak at 7.29 ppm corresponds to the natural abundance of deuterium in the CHCl_3_ solvent.

**Figure S5.** FT-IR spectra of crystalline and amorphous (a) **Cu_18_-1** and (b) **Cu_18_-2**.

**Figure S6.** UV-vis absorption spectra of **Cu_18_-1** and **Cu_18_-2** with corresponding *E*_g_.

**Figure S7.** SEM image and corresponding elemental mapping images of the **Cu_18_-1** crystals. (a) SEM image of single crystal; Elemental mapping images of (b) Cu, (c) P elements, respectively; (d) EDS spectrum confirming the presence of above elements (Cu, P) in **Cu_18_-1**.

**Figure S8.** SEM image and corresponding elemental mapping images of the **Cu_18_-2** crystals. (a) SEM image of single crystal; Elemental mapping images of (b) Cu, (c) P elements, respectively; (d) EDS spectrum confirming the presence of above elements (Cu, P) in **Cu_18_-2**.

**Figure S9.** XPS survey scan spectra of (a) **Cu_18_-1** and (d) **Cu_18_-2**. Core-level XPS spectra of the Cu 2p electrons in (b) **Cu_18_-1** and (e) **Cu_18_-2**. Cu LMM Auger spectra of (c) **Cu_18_-1** and (f) **Cu_18_-2**. The binding energy was calibrated based on the C 1s peak at 284.8 eV.

**Figure S10.** The thermal ellipsoids of the ORTEP diagram of the **Cu_18_-1**.

**Figure S11.** The thermal ellipsoids of the ORTEP diagram of the **Cu_18_-2**.

**Figure S12.** (a) Top and (b) side views of the **Cu_18_-1** crystal structure, showing complete overlap after 90° rotation along the ***C*_4_** axis. Color labels: orange = Cu; dark blue = P; grey = C; light blue = H_lattice_; white = H.

**Figure S13.** Unit cell plots of (a) **Cu_18_-1** and (b) **Cu_18_-2**. Color labels: orange = Cu; blue = P; light green = F; light pink = B; green = Cl; grey = C; white = H.

**Figure S14.** Packing mode of **Cu_18_-1** in the crystal shown. (a) Along a axis; (b) along b axis; (c) along c axis. All non-metallic atoms are omitted for clarity. The **Cu_18_-1** molecules arranged in different directions show different colors (red/ blue). Color labels: green = Cl.

**Figure S15.** Packing mode of **Cu_18_-2** in the crystal shown. (a) Along a axis; (b) along b axis; (c) along c axis. All non-metallic atoms are omitted for clarity. The **Cu_18_-2** molecules arranged in different directions show different colors (red/ blue). Color labels: light green = F; light pink = B.

**Figure S16.** Distribution of H on the Cu_5_H_4_ pyramid, (a) top view and (b) side view.

**Figure S17.**The interpenetrating connection of L3 and L4 forms Cu_8_H_8_ unit, (a) top view and (b) side view.

**Figure S18.** Bond lengths analysis of the (a) **Cu_18_-1** and (b) **Cu_18_-2**. Color labels: orange = Cu; blue = P.

**Figure S19.** Exploded view of electrocatalytic reactors tested for eCO_2_RR in flow cell.

Figure S20. Gas products (CO, CH_4_ and C_2_H_4_) analysis for eCO_2_RR on (a) Cu_18_-1/GDE and (b) Cu_18_-2/GDE catalysts.

**Figure S21.** Liquid products (formate, EtOH and CH_3_COOH) analysis for eCO_2_RR on (a) **Cu_18_-1/GDE** and (b) **Cu_18_-2/GDE** catalysts.

**Figure S22.** Tafel plots constructed for the eCO_2_RR on the **Cu_18_-1/GDE** and **Cu_18_-2/GDE** catalysts.

**Figure S23.** Electrochemical impedance spectra of the **Cu_18_-1/GDE** and **Cu_18_-2/GDE** catalysts.

**Figure S24.** Reduction product distribution obtained on (a) the **Cu_18_-1/GDE** and (b) **Cu_18_-2/GDE** catalysts, respectively.

**Figure S25.** Selectivity for C_2_H_4_, H_2_ and other C-containing products obtained on (a) **Cu_18_-1/GDE** and (b) **Cu_18_-2/GDE** catalysts.

**Figure S26.** *FE*s of various eCO_2_RR products obtained on **Cu_18_-1/GDE** and **Cu_18_-2/GDE** catalysts. (a) H_2_; (b) CO; (c) CH_4_; (d) liquid products (formate, EtOH, and CH_3_COOH).

**Figure S27.** *J* of various eCO_2_RR products obtained on **Cu_18_-1/GDE** and **Cu_18_-2/GDE** catalysts. (a) H_2_; (b) CO; (c) CH_4_; (d) liquid (formate, EtOH, and CH_3_COOH).

**Figure S28.** The thermal ellipsoid plot and structure of **Cu_18_-1-BF_4_**.

**Figure S29**. **(**a) UV-vis absorption spectrum and (b) ESI-MS (in positive mode) of **Cu_18_-1-BF_4_**, with experimental and simulated isotopic patterns.

**Figure S30.** SEM image and corresponding elemental mapping images of the **Cu_18_-1-BF_4_** crystals. (a) SEM image of single crystal; Elemental mapping images of (b) Cu, (c) P, (d) B, (e) F elements, respectively; (f) EDS spectrum confirming the presence of above elements (Cu, P, B, F) in **Cu_18_-1-BF_4_**.

**Figure S31.** (a) The reduction product distribution and (b) *J*_C2H4_ on **Cu_18_-1-BF_4_/GDE**.

**Figure S32.** (a) Product signals detected by gas mass spectrometry after the reduction of ^13^CO_2_. (b) Time-dependent signal profiles of various ^13^C-labeled gases.

**Figure S33.** Cyclic voltammetry of (a) **Cu_18_-1/GDE** and (c) **Cu_18_-2/GDE** catalysts at a scan rate from 20, 40, 60, 80 and 100 mV·s^–1^; The corresponding linear curves of current density and scan rate of (b) **Cu_18_-1/GDE** and (d) **Cu_18_-2/GDE** catalysts.

**Figure S34**. Long-term stability test of C_2_H_4_ production on **Cu_18_-2** was conducted at −1.5 V_RHE_ for 120 h.

**Figure S35.** ^1^H-NMR spectra of the EtPP phosphine ligand and Cu_18_ NCs before and after eCO_2_RR.

**Figure S36.** ESI-MS (positive mode) and HR-TEM image and corresponding particle size distribution of the (a, b) **Cu_18_-1** and (c, d) **Cu_18_-2** after 120 h of electrolysis.

**Figure S37.** eCO_2_RR performance in the catalyst dissolution-redeposition experiment. *FEs* obtained on different working electrodes: (a) bare GDE; (b) electrode after DCM extraction, containing only residual Cu species; and (c) fresh GDE re-coated with the DCM extract recovered from the post-eCO_2_RR electrode. ICP analysis shows that the decomposition loss of the NCs for only 2.19%.

**Figure S38.** Core-level XPS spectra of the Cu 2p electrons in **Cu_18_-1/GDE** and **Cu_18_-2/GDE** before and after eCO_2_RR.

**Figure S39.** High resolution transmission electron microscope (HR-TEM) images of **Cu_18_-1/GDE** before and after eCO_2_RR.

**Figure S40.** Proposed ligand-stripping pathways for (a) **Cu_18_-1** and (b) **Cu_18_-2**. Exposed active metal areas of (c) **Cu_18_-1** and (d) **Cu_18_-2** after removal of one or two phosphine ligands. Blue and black phosphine ligands denote those involved and not involved in intramolecular interactions, respectively.

Guided by the crystallographically resolved intramolecular interactions and post-electrolysis ESI-MS results, the ligand-removal sites used in the DFT models were assigned in a structure-dependent manner. In **Cu_18_-1**, six EtPP ligands are involved in intramolecular interactions, whereas three of the four noninteracting EtPP ligands are spatially clustered on one side of the cluster (Fig. S40a). Because ESI-MS reveals the loss of two EtPP ligands from **Cu_18_-1** under eCO_2_RR conditions, these adjacent noninteracting EtPP ligands were selected as the most plausible ligand-stripping sites, giving rise to a relatively large exposed active-site window of ca. 11.2 Å (Fig. S40c). In contrast, all TPP ligands in **Cu_18_-2** participate in intramolecular interactions, consistent with the ESI-MS observation that only one TPP ligand dissociates after electrolysis (Fig. S40b). Accordingly, the TPP ligand associated with the weakest intramolecular interaction was chosen as the most likely dissociation site, generating a smaller exposed active-site window of ca. 7.6 Å (Fig. S40d). Notably, the selected TPP dissociation site in **Cu_18_-2** is spatially analogous to the EtPP dissociation site in **Cu_18_-1**, enabling a direct comparison of ligand-stripping energetics between the two clusters.

Section 3. Supporting Table

**Table S1** The crystal structure parameters for [Cu_18_H_17_(EtPP)_10_]Cl**.**

| Empirical formula | C_140_H_150_ClCu_18_P_10_ |
| --- | --- |
| Formula weight | 3321.46 |
| Temperature/K | 296.15 K |
| Crystal system | triclinic |
| Space group | *P-1* |
| a/Å | 16.711(2) |
| b/Å | 21.294(2) |
| c/Å | 22.471(3) |
| α/° | 90.463(2) |
| β/° | 93.450(3) |
| γ/° | 103.804(3) |
| Volume/Å^3^ | 7749.2(15) |
| Z | 2 |
| ρ_calc_g/cm^3^ | 1.423 |
| μ/mm^‑1^ | 2.574 |
| F(000) | 3358.0 |
| Radiation | Mo Kα (λ = 0.71073) |
| 2Θ range for data collection/° | 3.89 to 49.996 |
| Index ranges | -19 ≤ h ≤ 19, -25 ≤ k ≤ 25, -26 ≤ l ≤ 26 |
| Reflections collected | 222266 |
| Independent reflections | 27219 [R_int_ = 0.1641, R_sigma_ = 0.0844] |
| Data/restraints/parameters | 27219/1179/1511 |
| Goodness-of-fit on F^2^ | 0.961 |
| Final R indexes [I>=2σ (I)] | R_1_ = 0.0553, wR_2_ = 0.1414 |
| Final R indexes [all data] | R_1_ = 0.1147, wR_2_ = 0.1637 |
| Largest diff. peak/hole / e Å^-3^ | 0.95/-0.51 |

**Table S2** The crystal structure parameters for [Cu_18_H_17_(EtPP)_10_]BF_4_.

| Empirical formula | C_140_H_150_BCu_18_F_4_P_10_ |
| --- | --- |
| Formula weight | 3372.82 |
| Temperature/K | 210 |
| Crystal system | triclinic |
| Space group | *P-1* |
| a/Å | 16.6641(7) |
| b/Å | 21.2507(13) |
| c/Å | 22.4381(13) |
| α/° | 89.675(2) |
| β/° | 86.4400(10) |
| γ/° | 75.525(2) |
| Volume/Å^3^ | 7678.4(7) |
| Z | 2 |
| ρ_calc_g/cm^3^ | 1.459 |
| μ/mm^‑1^ | 2.585 |
| F(000) | 3406.0 |
| Radiation | MoKα (λ = 0.71073) |
| 2Θ range for data collection/° | 3.41 to 50.5 |
| Index ranges | -19 ≤ h ≤ 19, -25 ≤ k ≤ 25, -26 ≤ l ≤ 26 |
| Reflections collected | 106676 |
| Independent reflections | 27742 [R_int_ = 0.1040, R_sigma_ = 0.0925] |
| Data/restraints/parameters | 27742/1492/1755 |
| Goodness-of-fit on F^2^ | 0.988 |
| Final R indexes [I>=2σ (I)] | R_1_ = 0.0490, wR_2_ = 0.1173 |
| Final R indexes [all data] | R_1_ = 0.0761, wR_2_ = 0.1276 |
| Largest diff. peak/hole / e Å^-3^ | 0.83/-0.72 |

**Table S3** Comparison of electrocatalytic CO_2_-to-C_2_H_4_ conversion activity among reported Cu NCs and Cu-based metal-organic complex electrocatalysts.

| **Sample** | **Electrolyte** | **Electrolyzer** | **Highest *FE*_C2H4_**  **(%)** | ***j*_C2H4_**  **(mA·cm^-2^)** | **Ref** |
| --- | --- | --- | --- | --- | --- |
| Cu_18_-1 | 0.5M KOH + 0.5M KCl | Fllowcell | 70.59 (at -1.4 V) | 298.62 | This work |
| Cu_18_-2 | 0.5M KOH + 0.5M KCl | Fllowcell | 44.28 (at -1.5 V) | 148.07 | This work |
| Cu_17_ | 1M KOH | Fllowcell | ~40.00 (at -1.1 V) | ~37.50 | [1] |
| Cu_38_ | 1M KOH | Fllowcell | ~39.00 (at -1.4 V) | ~35.00 | [2] |
| Cu_4_ | 1M KOH | Fllowcell | ~13.00 (at -1.5 V) | — | [3] |
| Cu_8_ | 1M KOH | Fllowcell | ~37.00 (at -1.2 V) | — | [3] |
| Cu_6_ | 1M KOH | Fllowcell | ~25.00 (at -1.4 V) | ~64.40 | [4] |
| Cu_45_ | 1M KOH | Fllowcell | ~58.00 (at -1.6 V) | ~118.00 | [5] |
| Cu_67_ | 1M KOH | Fllowcell | 39.01 (at -0.8 V) | ~40.00 | [6] |
| Cu_4_@Ti_9_ | 1M KOH | Fllowcell | ~47.6 (at -1.0 V) | 400.00 | [7] |
| Inz-Cu_3_ | 1M KOH | Fllowcell | 35.27% (at -0.9 V) | — | [8] |
| Cu dimer-distorted  CuBTC | 1M KOH | Fllowcell | 45% (at 1.1 V) | 117.9 | [9] |
| Cu(OH)BTA | 1M KOH | Fllowcell | 57% (at -0.9 V) | 285 | [10] |

**Reference**

[1]	Han, B. L.; Zhao, L. C.; Yuan, Z. R.; Wang, Z.; Yu, Q.; Luo, G. G.; Wang, L. K.; Tung, C. H.; Sun, D.*,* *Adv. Funct. Mater.* **2025**, *35*, 2500149.

[2]	Mu, W.-L.; Li, L.; Cong, X.-Z.; Chen, X.; Xia, P.; Liu, Q.; Wang, L.; Yan, J.; Liu, C.*,* *J. Am. Chem. Soc.* **2024**, *146*, 28131-28140.

[3]	Li, J.-K.; Dong, J.-P.; Liu, S.-S.; Hua, Y.; Zhao, X.-L.; Li, Z.; Wang, R.; Zhao, S.-N.; Zang, S.-Q.*,* *Angew. Chem. Int. Ed.* **2024**, *63*, e202412144.

[4]	Wu, Q.-J.; Si, D.-H.; Sun, P.-P.; Dong, Y.-L.; Zheng, S.; Chen, Q.; Ye, S.-H.; Sun, D.; Cao, R.; Huang, Y.-B.*,* *Angew. Chem. Int. Ed.* **2023**, *62*, e202306822.

[5]	Zhang, B.; Long, Z. C.; Xu, J. R.; Xu, H. Y.; Wang, Q. M.*,* *J. Am. Chem. Soc.* **2026**, *148*, 2519-2528.

[6]	Liu, Z. Y.; Li, S. Q.; Zhao, L. C.; Qin, L. B.; Yang, J. W.; Wu, T.; Wang, L. K.; Tang, Q.; Tang, Z. H.*,* *Nano Res.* **2026**, *19*, 94908145.

[7]	X. Fan, J. Cheng, M. Qiu, Y. F. Zhang, S. Chen, Z. J. Zhang, Y. Peng, J. Zhang and L. Zhang, *ACS Mater. Lett.*, **2023**, *5*, 1527-1531.

[8]	Wang, R.; Dong, L. Z.; Shi, J. W.; Zhang, M.; Li, S. L.; Lan, Y. Q.; Liu, J.*,* *Acs Catal.* **2024**, *14*, 741-750.

[9]	Nam, D. H.; Bushuyev, O. S.; Li, J.; De Luna, P.; Seifitokaldani, A.; Dinh, C. T.; Garcia de Arquer, F. P.; Wang, Y.; Liang, Z.; Proppe, A. H.; Tan, C. S.; Todorovic, P.; Shekhah, O.; Gabardo, C. M.; Jo, J. W.; Choi, J.; Choi, M. J.; Baek, S. W.; Kim, J.; Sinton, D.; Kelley, S. O.; Eddaoudi, M.; Sargent, E. H.*,* *J. Am. Chem. Soc.* **2018**, *140*, 11378-11386.

[10]	Liang, Y.; Zhao, J.; Yang, Y.; Hung, S. F.; Li, J.; Zhang, S.; Zhao, Y.; Zhang, A.; Wang, C.; Appadoo, D.; Zhang, L.; Geng, Z.; Li, F.; Zeng, J.*,* *Nat. Commun.* **2023**, *14*, 474.
